# Systemic Inflammatory Response Syndrome, Thromboinflammation, and Septic Shock in Fetuses and Neonates

**DOI:** 10.3390/ijms26073259

**Published:** 2025-04-01

**Authors:** Victoria Bitsadze, Arina Lazarchuk, Alexander Vorobev, Jamilya Khizroeva, Maria Tretyakova, Natalia Makatsariya, Nilufar Gashimova, Kristina Grigoreva, Alena Tatarintseva, Anna Karpova, Aleksei Mostovoi, Marina Zainulina, Daredzhan Kapanadze, Armen Blbulyan, Nart Kuneshko, Jean-Christophe Gris, Ismail Elalamy, Grigoris Gerotziafas, Alexander Makatsariya

**Affiliations:** 1 Department of Obstetrics, Gynecology and Perinatal Medicine, The I.M. Sechenov First Moscow State Medical University (Sechenov University), Trubetskaya Str 8-2, Moscow 119435, Russia; vikabits@mail.ru (V.B.); lazarchuk_a_v@student.sechenov.ru (A.L.); alvorobev@gmail.com (A.V.); tretyakova777@yandex.ru (M.T.); makatsariya@gmail.com (N.M.); nelya.94@yandex.ru (N.G.); grigkristik96@gmail.com (K.G.); tatarintseva@mail.ru (A.T.); jean.christophe.gris@chu-nimes.fr (J.-C.G.); ismail.elalamy@aphp.fr (I.E.); grigorios.gerotziafas@inserm.fr (G.G.); gemostasis@mail.ru (A.M.); 2 Vorokhobov City Clinical Hospital No 67, Moscow Healthcare Department, 2/44 Salyama Adilya Str., Moscow 123423, Russia; anna1409@mail.ru (A.K.); valmost@mail.ru (A.M.); 3 Russian Medical Academy of Continuous Professional Education, Health Ministry of Russian Federation, 2/1 bldg. 1, Barrikadnaya Str., Moscow 123993, Russia; 4 Department of Polyclinic Therapy, Clinical Laboratory Diagnostics and Medical Biochemistry of Institute of Postgraduate Education of Yaroslavl State Medical University, Yaroslavl State Medical University, Health Ministry of Russian Federation, 5 Revolutsionnaya Str., Yaroslavl 150000, Russia; 5 Snegirev Maternity Hospital No 6, 5 Mayakovskogo Str., Saint Petersburg 192014, Russia; zainulina@yandex.ru; 6 Department of Obstetrics, Gynecology and Reproductology of Pavlov First Saint Petersburg State Medical University, Pavlov First Saint Petersburg State Medical University, Health Ministry of Russian Federation, 6/8 Lev Tolstoy Str., Saint Petersburg 197022, Russia; 7 Center of Pathology of Pregnancy and Hemostasis «Medlabi», 340112 Tbilisi, Georgia; medlabimedlabi@gmail.com; 8 Research Center of Maternal and Child Health Protection, 22 Mashtots Avenue, Yerevan 0002, Armenia; belbulyan@mail.ru; 9 Moscow’s Region Odintsovo Maternity Hospital, Odintsovo 143003, Russia; drnartfaruk@mail.ru; 10 Faculty of Pharmaceutical and Biological Sciences, Montpellier University, 34093 Montpellier, France; 11 Faculté Privee de Médecine de Marrakech (FPMM), Route Amizmiz, Marrakech 42312, Morocco; 12 Hopital Americain de Paris, 55 rue du Château, Neuilly Sur Seine, 92200 Paris, France; 13 INSERM UMR_S_938, Saint-Antoine Research Center (CRSA), Team “Cancer Biology and Therapeutics”, Group “Cancer—Angiogenesis—Thrombosis”, University Institute of Cancerology (UIC), Sorbonne University, 34 Rue du Crozatier, 75012 Paris, France; 14 Thrombosis Center, Tenon—Saint Antoine University Hospital, Hôpitaux Universitaires Est Parisien, Assitance Publique Hôpitaix de Paris (AP-HP), 4 Rue de la Chine, 75020 Paris, France

**Keywords:** systemic inflammatory response syndrome, thromboinflammation, septic shock, fetal inflammatory response syndrome

## Abstract

This article explores systemic inflammatory response syndrome (SIRS), thromboinflammation, and septic shock in fetuses and neonates, offering a comprehensive examination of their pathophysiology, diagnostic criteria, and clinical implications. It identifies SIRS as an exaggerated response to external stress, disrupting the balance between inflammation and adaptive mechanisms, driven by cytokines such as TNF-α and IL-1. The fetal inflammatory response syndrome (FIRS), a subset of SIRS, is noted for its role in adverse neonatal outcomes, including organ damage, inflammation, and long-term developmental disorders. The article discusses the extensive effects of FIRS on critical systems, including the blood, lungs, central nervous system, and kidneys. It highlights the challenges in diagnosing and managing septic shock in neonates, focusing on the relationship between inflammation and the hemostatic system. Additionally, the paper points out recent advancements, such as the convergent model of coagulation and emerging biomarkers like microRNAs for early detection. Despite this progress, gaps remain in understanding the molecular mechanisms underlying these conditions and in developing effective therapeutic strategies. This highlights the necessity for targeted research to mitigate the morbidity and mortality associated with septic shock in neonates.

## 1. Introduction

Neonatal sepsis and septic shock remain significant health issues globally, serving as a primary cause of mortality and morbidity among newborns. Shock is an unstable and dynamic pathophysiological condition marked by insufficient tissue perfusion. Extended hypoperfusion and tissue hypoxia may interrupt essential biochemical processes, and if not corrected, this can lead to organ failure and death.

In 1894, the distinguished Italian biologist Giuseppe Sanarelli replicated the condition of septic (endotoxin) shock in an animal experiment. If there were no noticeable clinical manifestations of the disease after the first sublethal dose of typhoid toxin administered to rabbits, then a second dose of the same toxin given 24 h later resulted in the animal’s death. In 1926, another scientist, Gregory Schwartzman from Mount Sinai Hospital in New York, experimentally reproduced the reaction and connected it to the direct effects of bacterial toxins on the vascular endothelium, leukocytes, thrombocytes, and serum defense systems. This phenomenon later became known as the Sanarelli–Schwartzman phenomenon and is defined as a localized or generalized response of the body to endotoxins, which induce thrombosis and ultimately lead to necrosis of the affected tissues.

Unfortunately, the term “Sanarelli–Schwartzman phenomenon” has become less common among doctors over time—clinicians have forgotten about it. However, under other broad categories, the Sanarelli–Schwartzman phenomenon is well known to all clinicians, such as systemic inflammatory response syndrome (SIRS), disseminated intravascular coagulation (DIC), septic shock, fulminant purpura, hemolytic–uremic syndrome, and Waterhouse–Friderichsen syndrome.

In fact, the generalized response of Sanarelli–Schwartzman serves as a model for sepsis and septic shock. This phenomenon involves the activation of the clotting system, leading to consumption coagulopathy and intravascular clotting within the microcirculatory system. In 2020, the names of Grigory Schwartzman and Giuseppe Sanarelli regained prominence in the scientific community in the context of the COVID-19 pandemic [1].

## 2. Systemic Inflammatory Response Syndrome, Thromboinflammation, and Septic Shock in Fetuses and Neonates

### 2.1. Systemic Inflammatory Response Syndrome, SIRS

SIRS is a hyper-response of the body to an external stressor, which may include inflammation from an infectious process, surgical intervention, or malignancy [2]. The regulation of the body’s response should effectively suppress the source of inflammation; however, in the development of SIRS, the balance between inflammation and the adaptive anti-inflammatory mechanism is disrupted [3].

#### 2.1.1. Diagnostic Criteria for SIRS

Table 1 presents the key diagnostic criteria for SIRS. Two or more criteria indicate that SIRS has developed [4].

#### 2.1.2. The Pathogenesis of SIRS

The pathogenesis of SIRS is based on the production of pro-inflammatory cytokines, particularly TNF-α (tumor necrosis factor), IL-1 (interleukin-1), IL-6 (interleukin-6), IL-8 (interleukin-8), and IL-10 (interleukin-10), which spread to sites distant from the primary focus. The inflammatory response, initiated via the release of inflammatory mediators, is a defensive mechanism essential for tissue repair and regeneration following injury, ischemia, or infection. However, complex mechanisms that precisely regulate the intensity and duration of this response are crucial because uncontrolled and dysregulated inflammatory responses can result in tissue damage and act as the underlying mechanism of chronic inflammation diseases. In this case, TNF-α and IL-1, which stimulate tissue factor (TF) production, play a major role in the development of endothelial dysfunction. As a result of systemic effects on the endothelium and changes in the balance between the coagulation system and fibrinolysis, the whole hemostasis system is disturbed, leading to the development of organ failure. Stimulation of the coagulation system and suppression of fibrinolysis results in increased thrombosis, impaired microcirculation, and further organ damage.

Cytokines also activate MAPK/NF-κB (mitogen-activated protein kinase/ nuclear transcription factor kappa-B) pathways within endothelial cells. These pathways have been considered a central mediator of inflammation and an important bridge in the switch from extracellular signals to intracellular responses. MAPK/NF-κB pathways can disrupt inflammatory processes in various ways, leading to increased synthesis and release of procoagulant factors [5].

SIRS is an overwhelming inflammatory response of the body to an infectious or non-infectious trigger characterized by the uncontrolled release of excessive pro-inflammatory modulators. It can result in endothelial dysfunction, microvascular thrombosis, and organ damage. In recent years, research has increasingly highlighted the importance of SIRS in the development of serious pregnancy complications, including recurrent pregnancy loss, preterm delivery, placenta accreta, and fetal growth restriction (FGR) preeclampsia [6].

### 2.2. Fetal Inflammatory Response Syndrome, FIRS

The condition known as fetal inflammatory response syndrome (FIRS) refers to a systemic inflammatory reaction in the fetus, is associated with infectious and inflammatory changes in the placenta, and is linked to neonatal morbidity and mortality. The variety of microorganisms that can infect the fetus and the necessity for various diagnostic methods complicate pathogen identification, particularly in high-risk neonates. Several studies indicate that the presence of intrauterine infection during pregnancy is associated with a significant risk of serious long-term consequences. These consequences include exudative otitis media and neuroendocrine-immune dysfunction in the child [7,8]. Therefore, it is particularly important to address intrauterine infection from the perspective of FIRS. Despite the high morbidity and mortality rates, there is a lack of convincing data on early diagnosis and effective treatment for this pathological condition.

The molecular mechanisms of FIRS are still not fully understood. However, it has been proposed to categorize this syndrome into two types based on differences in the inflammatory response. Type 1 FIRS is characterized by acute inflammation and elevated levels of IL-6, IL-8, and CXCL10 (C-X-C motif chemokine ligand 10, a chemokine whose levels rise in the amniotic fluid of patients with chronic chorioamnionitis), IL-1β (Interleukin-1β), and TNF-α compared to healthy newborns. Additionally, significant changes in gene expression occur in FIRS type 1, leading to the suppression of T-cell immune regulation [9]. Thymic involution, often seen in newborns with acute inflammation, explains this response [10]. Straňák et al., after analyzing the cord blood of 100 preterm infants, also discovered a correlation between the presence of FIRS and elevated levels of IL-6, CRP (C-reactive protein), and procalcitonin. Maternal leukocytosis (*p* < 0.001), premature rupture of membranes (*p* < 0.001), and preterm uterine contractions (*p* < 0.0001) were observed in relation to FIRS type 1 [11].

In contrast, there is no acute inflammatory response in FIRS type 2. However, there is upregulation of HLA-F (major histocompatibility complex, class I, F), HLA-C (major histocompatibility complex, class I, C), and HLA-DRA (major histocompatibility complex, class II, DR alpha) genes, along with a significant increase in CXCL10 and a mild chronic inflammatory response, resembling the mechanisms of graft rejection [12].

### 2.3. Multiple Organ Failure in FIRS

The foundation of FIRS is the maternal inflammatory response (MIR), which includes acute subchorionitis, inflammation of the placenta, and inflammation of the extraplacental membranes. The diagnostic criteria for FIRS comprise the fetal inflammatory reaction (FIR), acute inflammation of the placenta, extraplacental membranes, umbilical cord, and chorionic vasculitis. Although FIR and MIR may be absent in patients with FIRS type 2, this does not diminish the risk of subsequent neonatal complications [13]. In the context of FIRS, the fetus exhibits signs of damage to multiple organs and body systems. The most affected organs include the blood, lungs, central nervous system, thymus, spleen, and kidneys. Additionally, fetal inflammation is associated with necrotizing enterocolitis and cerebral hemorrhage.

The hematological changes in fetuses with FIRS indicate a trend of elevated neutrophil counts and increased erythropoiesis. As demonstrated by Romero et al., fetuses with FIRS were more likely to have neutrophil counts exceeding the 95th percentile for gestational age (71% (30/42) vs. 35% (37/105); *p* < 0.001) [14]. One mechanism responsible for fetal neutrophilia is the increase in granulocyte colony-stimulating factor (G-CSF), a protein known for its ability to stimulate the production of various cells, including neutrophils, macrophages, eosinophils, T-helper cells (Th1 and Th17), and certain tumor cells. The expression of G-CSF is enhanced by inflammatory mediators such as IL-1, IL-4, IL-6, and TNF-α but can be inhibited by several cytokines, including IL-10 and interferon-gamma [15,16]. Furthermore, G-CSF is recognized for promoting the release of neutrophils in response to stressful conditions like infectious processes. Chaiworapongsa et al. demonstrated that G-CSF levels were significantly elevated in fetuses with FIRS compared to controls (*p* < 0.001). They also identified that a fetal plasma G-CSF concentration of ≥134 pg/mL was a significant predictor of chorioamnionitis, infection, and cumulative neonatal morbidity and mortality [17]. An increased number of nucleated erythrocytes (immature forms) in neonates has been observed in cases of prolonged rupture of fetal membranes (>24 h), with a histological diagnosis of chorioamnionitis and early-onset neonatal sepsis. These observations indicate an increase in erythropoiesis [18,19]. It is commonly assumed that circulating nucleated erythrocytes are a reliable indicator of hypoxia; however, in the case of FIRS, no acidosis or hypoxemia was observed. Additionally, the pH (−0.026 and −0.016), PaO(2) (0.25 mmHg and 5.9 mmHg), and BE (−2.4 and −2.6 mEq/L) values did not differ significantly between the fetuses without FIRS and those affected by it (*p* < 0.05) [20].

Zaharie et al. studied the dynamics of plasma levels of key pro-inflammatory cytokines (TNF-α, IL-6, and neutrophil-activating peptide 78 (ENA-78)) and the anti-inflammatory cytokine IL-10 on the first and third days of life in 80 neonates at the tertiary Neonatal Intensive Care Unit in Cluj-Napoca, Romania, as well as the correlation between these levels and neonatal morbidity and mortality [21]. pH, oxygen saturation, the fraction of inhaled oxygen (FiO2), the gestational age at which the premature rupture of the fetal membranes (PROM) occurred, and venous blood cytokine levels were assessed. The results demonstrated that neonates born to mothers with PROM exhibited elevated levels of all cytokines. Symptoms of necrotizing enterocolitis (NEC) were associated with elevated levels of IL-6, while the development of cerebral intraventricular hemorrhage in neonates correlated with high CXCL-5 (*p* = 0.037) and sepsis was associated with high IL-10 levels (*p* = 0.02). The decrease in ENA-78 (*p* = 0.026) and IL-10 (*p* < 0.001) was significant from the first day to the third day, which enhanced the survival rate and indicated the conclusion of the inflammatory process.

The lungs represent a crucial target organ in fetuses with FIRS. Elevated levels of IL-6 in cord blood are associated with an increased risk of bronchopulmonary dysplasia (BPD) in neonates. Kallapur et al. demonstrated that administering intra-amniotic endotoxin in vivo results in an enhanced inflammatory response and multiple increases in mRNA levels of IL-1, IL-6, IL-8, and TNF-α in subsequent bronchoalveolar lavage [22]. Administering endotoxin in vivo leads to an increase in surfactant synthesis and structural changes in the fetal lungs, including an enlarged alveolar diameter and thinned alveolar septa. These changes facilitate accelerated lung maturation and preparation for preterm labor [23,24]. Yoon et al. demonstrated the correlation between IL-6 levels in cord blood and amniotic fluid and the subsequent development of BPD in 203 patients. The results revealed that 17% of the patients’ exhibited symptoms of BPD. The findings indicated that IL-6 levels in cord blood were significantly elevated in newborns with BPD (OR 4.2; 95% CI 1,6–11,2) [25]. However, a meta-analysis by Jackson et al. found no correlation between BPD and chorioamnionitis when adjusted for gestational age (OR 0.99, CI 0.76–1.3). As a result, prospective studies will be necessary to determine the pathogenetic mechanisms underlying these conditions [26].

Other complications during the perinatal period in children include respiratory distress syndrome (RDS), which arises from increased cortisol secretion and surfactant deficiency, as well as persistent pulmonary hypertension. The symptom complex of neonatal RDS encompasses cyanosis, tachypnea, retraction of the flexible areas of the chest, and a distinct wheeze that occurs when exhaled air enters the partially closed vocal tract during or shortly after birth [27]. In the study by Dessardo et al., the presence of FIRS was identified as the most significant risk factor for both chronic lung disease of prematurity (OR 31.05, 95% CI 10.7–87.75, *p* < 0.001) and wheezing in infants (OR 5.63, 95% CI 2.42–13.05, *p* = 0.01) [28].

Several authors have suggested that fetal inflammatory response syndrome (FIRS) impacts brain function and leads to neuroinflammation [29]. The pathogenic mechanism behind brain damage is not yet fully understood and is likely related to the “multiple hits” theory. Chorioamnionitis causes widespread damage to both white and gray matter neurons, primarily due to the direct harmful effects of pro-inflammatory cytokines (TNF-α, IL-1, and IL-6) [30,31,32]. Additionally, inflammatory mediators may influence the integrity of the blood–brain barrier (BBB), contributing to increased permeability of the BBB to various proteins and worsening brain damage [33]. The activation of microglia, which serves as the first line of defense for nerve cells during inflammation, plays a crucial role in the pathogenesis of brain disorders. When endotoxin was administered to animals, significant activation of microglia was observed, along with changes in the immunoreactivity of myelin basic protein and apoptosis of oligodendrocytes [34]. In 2023, Giovannini et al. demonstrated that the primary and most life-threatening condition in newborns following FIRS is cerebral hypoxia (CH) [35]. For this reason, it is recommended that these children undergo a GM ultrasound or GM MRI to detect focal and periventricular leukomalacia, as well as cystic changes [36]. The consequences of GM hypoxia may be irreversible, potentially leading to cerebral atrophy, which is characterized by decreased white matter volume and enlarged ventricles. Another significant complication of FIRS is neonatal encephalopathy (NE), which is marked by altered consciousness, seizures, impaired muscle tone, and the inability to initiate or maintain respiration. NE is associated with a high mortality rate and long-term disabilities, including cerebral palsy (CP), various cognitive impairments, and damage to vision and hearing. In turn, CP encompasses a range of motor and sensory impairments, along with intellectual disability, perceptual issues, behavioral disorders, and alterations in consciousness [37].

Chorioamnionitis is a significant risk factor for developing retinopathy of prematurity (ROP). This condition results from various lesions that disrupt neurovascular growth in the immature retina, potentially due to gestational maternal hypoxia. Elevated plasma IL-6 levels indicate the severity of ROP and can serve as a prognostic factor in the disease’s progression [38,39].

Gibson et al. analyzed the risk of developing various psychiatric disorders in nearly five thousand children with a history of FIRS [40]. The findings and statistical analyses confirmed that children with FIRS were more likely to be diagnosed with neuropsychiatric disorders (OR = 1.21, CI 95% [1.09, 1.35]), including autism spectrum disorder (OR = 1.35, 95% CI [1.08, 1.67]), attention deficit hyperactivity disorder (ADHD) (OR = 1.27, 95% CI [1.07, 1.49]), conduct disorder (OR = 1.50, 95% CI [1.24, 1.81]), and post-traumatic stress disorder (PTSD) (OR = 2.46, CI 95% [1.21, 5.04]). A seven-year observation period was used in this study to assess the long-term effects of FIRS on newborn infants. However, it is important to note that psychiatric disorders are not necessarily related to complications of the neonatal period.

Furthermore, the kidneys are often affected in fetuses with FIRS. The relationship between the amniotic fluid index (AFI), which relies on renal function and fetal urination, and the presence of intra-amniotic infection was examined. A positive culture result and elevated levels of matrix metalloproteinase-8 (>23 ng/mL), IL-6, and TNF-α were noted more frequently in patients with oligohydramnios (*p* < 0.05) [41,42]. Azpurua et al. demonstrated that children born to mothers with intra-amniotic inflammation exhibited elevated levels of IL-6 and urea nitrogen. However, there were no noticeable changes in renal vascular function as assessed via ultrasound Doppler ultrasonography [43]. In studies using animal models, chorioamnionitis resulted in a reduced number of renal tubules (*p* < 0.05), but no significant differences were found in body weight or the degree of inflammation in the kidneys [44,45]. Nonetheless, Muk et al. showed that inflammation in the amnion led to an increase in creatinine and microalbumin concentrations—markers of renal dysfunction. Furthermore, elevated levels of biomarkers indicative of renal damage (e.g., LRG1 (leucine-rich alpha-2-glycoprotein 1), KIM1 (kidney injury molecule-1), NGAL (Neutrophil Gelatinase–Associated Lipocalin), HIF1A (Hypoxia-inducible factor-1α), and CASP3 (Caspase 3)) were observed in kidney tissue. Therefore, further research is necessary to clarify the impact of FIRS on fetal immune activation and kidney function [46].

Furthermore, FIRS has a significant impact on the thymus, which is the most critical organ for fetal immune defense. A study by Kuypers et al. found a decrease in lymphocyte levels and a reduction in the cortico-medullary area of the thymus following the intra-amniotic injection of endotoxin. Twenty-four hours after administration, the levels of IL-6, IL-17 mRNA, and TLR4 mRNA were elevated, indicating the acute activation of the thymus [47]. In the context of inflammation, there is a notable shift in the composition of CD8+ T-lymphocytes, accompanied by the activation of CD4+ T-lymphocytes, as shown by an increased expression of CD25 (*p* = 0.0001), HLA-DR, and CD69 (*p* = 0.0003) [48,49].

Other organs and systems can also be affected in FIRS, including the spleen, liver, thyroid, and intestine. In FIRS, there is an alteration in splenic blood flow and a significant increase in TNF-α and CD3 expression, indicating an inflammatory response [50,51,52]. The presumed mechanisms of bowel damage in FIRS involve an increased TNF-α-mediated impairment of intestinal wall microcirculation and a decreased expression of vascular endothelial growth factor (VEGF) [53,54]. Chorioamnionitis also decreases liver function. In animal models, the administration of an endotoxin resulted in impaired lipid and glucose metabolism, increased alanine aminotransferase and aspartate aminotransferase levels, and a decreased total antioxidant status [55]. Furthermore, during endotoxin administration, the production of cytokines by Kupffer cells, such as TNF-α, IL-8, and IL-18, increases, which precedes intestinal inflammation. This indicates a possible influence of hepatic cytokines on the subsequent development of NEC [56].

Umbilical vessel funiculitis serves as a marker for FIRS, which occurs more frequently in preterm infants with elevated serum levels of intercellular adhesion molecule-1 (sICAM-1) [57]. A proposed correlation exists between FIRS and endothelial dysfunction, as a reduction in the systemic inflammatory response in patients coincided with an increase in sICAM-1 (Soluble intercellular adhesion molecule-1) levels and endothelial activation. Moreover, elevated sICAM-1 levels have been noted in neonates experiencing CNS depression syndrome. Further studies are necessary to determine the role of sICAM-1 as a predictor of long-term consequences associated with FIRS [58,59].

Few studies have definitively established the link between specific hemodynamic abnormalities and fetal growth restriction (FGR) development. Eloundou et al. assessed the impact of intrauterine inflammation on the hemodynamic and structural abnormalities of the placenta (vascular malperfusion) and fetal outcomes in vivo. Virchow’s triad postulates that thrombus formation occurs when three conditions are present: hypercoagulability (as in pregnancy), vascular injury, and slow blood flow. When the mice were exposed to the infectious agent, the following observations were noted: placental endotheliitis, a decreased volume and thickness of the placentas, reduced expression of CD31 and vimentin, hemodynamic changes in uterine and umbilical blood flow, and decreased fibrinogen levels in the placentas due to the consumption of clotting factors and thrombus formation. The third component of Virchow’s triad, stasis in blood flow, may result from placental thinning in conjunction with damage to the vascular network. Additionally, the fetuses displayed the activation of fetal microglia, likely linked to impaired nutrient passage and toxin excretion through the placenta [60].

FIRS in neonates is associated with a higher risk of neonatal sepsis [61]. In a study by Nomiyama et al., neonates with FIRS and MIR/FIR showed a higher prevalence of neonatal sepsis compared to those without FIRS and MIR/FIR (*p* < 0.001) [13].

**Figure 1:**
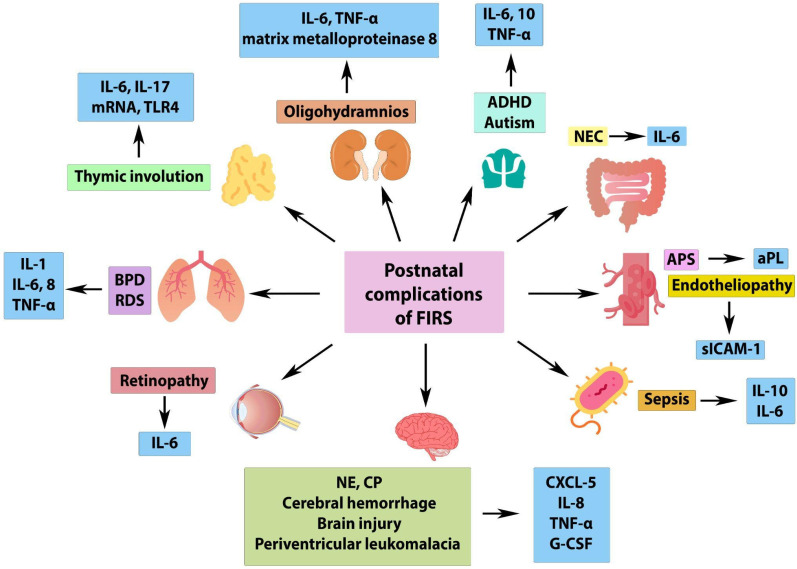
The image illustrates the postnatal complications of fetal inflammatory response syndrome (FIRS) and their associations with various inflammatory markers. It highlights neonatal conditions affecting multiple organs, including the brain, lungs, eyes, intestines, and immune system. ADHD—attention-deficit hyperactivity disorder, APS—antiphospholipid syndrome, CP—cerebral palsy, FIRS—fetal inflammatory response syndrome. TNF-α—tumor necrosis factor alpha; IL—interleukin; TLR4—toll-like receptor 4; NEC—necrotizing enterocolitis; BPD—bronchopulmonary dysplasia; RDS—respiratory distress syndrome; aPL—antiphospholipid antibodies; NE—neonatal encephalopathy; CP—cerebral palsy; sICAM-1—soluble intercellular adhesion molecule; CXCL5—C-X-C Motif Chemokine Ligand 5; G-CSF—granulocyte colony-stimulating factor.

The major complications of the neonatal period and associated cytokines after undergoing FIRS are summarized in Figure 1.

### 2.4. FIRS and Antiphospholipid Antibodies

It is important to note that the de novo synthesis of antiphospholipid antibodies (aPL) may be one of the responses to intra-amniotic inflammation when viewed from a “first-hit” perspective. As a result of inflammation during the perinatal period, there may be significant changes in the composition of the peripheral regulatory T-cell population in umbilical cord blood. Maternal infections may consequently lead to the reprogramming of the still immature innate and adaptive immune systems of the fetus [62]. The first three months of a newborn’s life are marked by the presence of maternal antibodies, which provide immunity. However, a year after birth, IgG and IgM levels achieve 50% of the total adult level. This may clarify the tendency for sepsis, septic shock, and other life-threatening conditions during the neonatal period, particularly in premature infants. However, the relationship between aPL and FIRS remains a topic of debate and ongoing study. Preterm infants with abnormal immune responses, along with the immaturity of most organs and systems (including the hemostatic system), face the highest risk of mortality and complications linked to inflammation and thrombosis [62]. It is increasingly clear that fetal immune shifts resulting from intrauterine infection and de novo aPL may significantly contribute to the development of neonatal thrombosis [62].

### 2.5. Perinatal Aspects of Septic Shock

In 2005, at the International Pediatric Sepsis Consensus Conference, specific definitions of pediatric SIRS, infection, sepsis, severe sepsis, and septic shock were established. Shock is a state of organ hypoperfusion characterized by cellular dysfunction. The pathogenesis of shock is diverse and includes several development mechanisms: a decrease in the circulating blood volume, reduced cardiac output, and vasodilation. Sepsis is a serious condition that arises from an excessive immune response to infection due to hematogenous and transplacental dissemination or due to vertical transmission of maternal infection, leading to organ dysfunction. The onset of sepsis in newborn infants is particularly perilous, correlating with increased mortality (OR 4.41, 95% CI 1.75–11.1) [63].

The development of sepsis can be attributed to various factors, categorized into several groups. These include maternal and neonatal risk factors [64]. (Table 2).

Sepsis can progress to septic shock, which is known for its high mortality rate among patients. Inadequate treatment of sepsis or its late diagnosis can lead to the spread of infection, which, in turn, results in generalized endothelial inflammation and subsequently septic shock. Statistical data indicate that septic shock develops in 10–15% of children with sepsis [65]. The high mortality rate seen in patients with septic shock is attributed to the nonspecific clinical manifestations and delayed diagnosis. Therefore, this condition requires particular attention from physicians.

The development of septic shock is a complex process involving immune system cells and the pathogen itself. Once in the bloodstream, the infectious agent is recognized by macrophages and monocytes through surface receptors, which initiates the activation of secondary messengers and intracellular cascades that promote the release of cytokines and chemokines. This is followed by the widespread activation of the endothelium, lymphocytes, and complement function, resulting in significant inflammation and shock [66].

The systemic spread of the pathogen and the generalized activation of the endothelium lead to impaired microcirculation and result in tissue hypoxia, acidosis, and hypotension. These factors contribute to the onset of septic shock in newborns [67]. The clinical manifestations of septic shock in neonates include damage to the respiratory and cardiovascular systems, along with various non-specific symptoms, such as decreased muscle tone and skin discoloration [68].

The clinical features of septic shock are characterized by three distinct stages of development, each with its own set of clinical characteristics:

(1) The initial phase, termed “compensated shock”, is characterized by the activation of neuroendocrine compensatory mechanisms [66]. The symptoms of stage 1 may include tachycardia, hypouresis, decreased tissue perfusion, and extremity coldness in the newborn.
(2) The subsequent stage in the development of septic shock is uncompensated shock, which is characterized by symptoms of systemic hypotension and metabolic acidosis.
(3) The final phase of septic shock development is irreversible shock, which is characterized by severe microcirculatory disorders and irreversible cellular damage, leading to necrosis and multi-organ failure.

Cold septic shock typically develops in children, characterized by changes in hemodynamics and increased systemic vascular resistance due to peripheral vasoconstriction. The symptom complex of cold septic shock includes decreased and weakened pulse, diuresis, and fingertip marbling. The onset of metabolic acidosis during the second stage of shock results in elevated pulmonary resistance, which is a crucial factor in the development of right ventricular failure.

### 2.6. Hemostasis in Newborns

The fetal and neonatal hemostatic systems differ significantly from those of adults. At first glance, neonates tend to bleed due to unique hemostatic properties, including prolonged activated partial thromboplastin time (aPTT), prothrombin time (PT), and delayed thrombin formation. However, despite these peculiarities, a healthy newborn does not experience an increased risk of bleeding or thrombosis. In healthy neonates, physiologically low levels of inhibitors can balance out low concentrations of clotting factors, thus maintaining the equilibrium of pro- and anticoagulant factors [69]. Factors that can induce bleeding include decreased platelet reactivity, pellet release, and levels of coagulation factors II, VII, IX, X, XI, and XII, as well as increased levels of alpha-2-macroglobulin. Conversely, factors that may cause thrombosis in neonates include increased hematocrit, mean corpuscular volume (MCV), von Willebrand factor (vWF), and decreased levels of protein S, protein C, and heparin cofactor II. In addition, thrombin production in neonates is about 90% of that observed in adults, which is sufficient for hemostatic clot formation [70].

Moreover, neonatal hemostasis has a limited buffer capacity, which raises the risk of thrombosis due to various acquired risk factors. These factors include multiple comorbidities, decreased fibrinolytic capacity, resistance to heparin arising from low antithrombin (AT) levels, and accelerated clearance of unfractionated heparin (UFH). The rising incidence of neonatal thrombosis may be associated with advancements in the treatment of life-threatening neonatal conditions, as well as a reduction in mortality among preterm infants. Additionally, platelet function and physiology depend on age. The study indicated an increase in fetal platelet count by roughly 2 × 10^9^/L for each week of gestation [71]. The mean platelet count in preterm infants was ≥200 × 10^9^/L, which is a normal level for adults. Normal platelet counts for newborn infants range from 150 × 10^3^ to 450 × 10^3^/μL. However, there is evidence that platelet counts in preterm infants may be lower than this value [72]. For this reason, preterm infants are more prone to developing hemorrhages, especially in the brain. The reactivity of platelets in neonates grows with age. A reduction in the expression of membrane glycoproteins in preterm infants leads to heightened platelet reactivity [73].

The hemostatic characteristics of newborns contribute to the development of non-specific clinical features of septic shock and SIRS.

### 2.7. Alterations in Neonatal Hemostasis in Septic Shock

**Figure 2:**
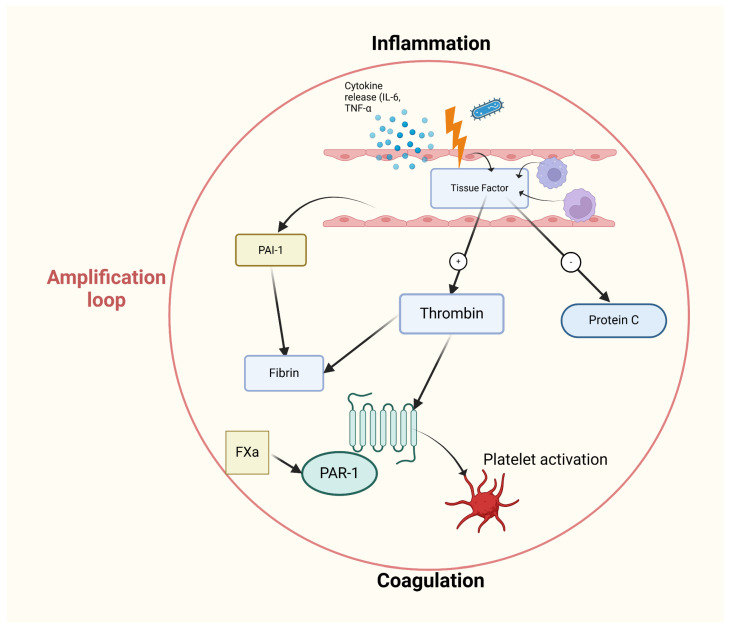
The relationship between inflammation and hemostasis—the “amplification loop.” Tissue factor activation leads to thrombin formation and platelet activation. This process contributes to a prothrombotic state, linking the immune response and clotting mechanisms. IL (Interleukin); TNF-α (Tumor necrosis factor); PAI-1 (Plasminogen activator inhibitor-1); FXa (Blood coagulation factor Xa); PAR-1 (Protease-activated receptor-1).

Pronounced disorders of hemostasis are common in patients with septic shock and can manifest in three distinct forms: chronic, subacute, and acute (disseminated intravascular coagulation, DIC). The relationship between inflammation and hemostasis is viewed through the lens of an “amplification loop”, in which inflammation initiates and sustains coagulation processes, while coagulation products further support and enhance inflammation [74] (Figure 2).

The activation of hemostasis in sepsis and septic shock occurs due to damage to the vascular endothelium. This damage is caused by pathogenic microorganisms, activated immune blood cells, and pro-inflammatory cytokines—particularly IL-6 and TNF-α—which lead to the production and release of tissue factor by monocytes, macrophages, and endothelial cells. In addition, tissue factor-mediated thrombin generation occurs, along with inhibition of protein C activity and fibrinolysis [75,76].

Another mechanism suggesting the presence of an “amplification loop” (Figure 2) is the interaction between protease-activated receptors (PARs) and coagulation factors, particularly TF-VIIa and factor Xa. This interaction activates intracellular signaling in endothelial cells, which also contributes to amplifying the inflammatory response. However, this mechanism has not been sufficiently studied in neonates [77].

The development of sepsis is associated with an increased release of plasminogen activator inhibitor type 1 (PAI-1) from endothelial cells, which inhibits plasmin activity. Bacteremia is linked to the early activation of fibrinolysis due to elevated levels of tissue-type plasminogen activator (tPA), resulting in a reciprocal release of PAI-1. However, the increase in tPA is not the only factor contributing to the rise in PAI-1 levels; the circulation of various cytokines, such as TNF-α and IL-6, also plays a role in this process. Elevated levels of TNF-α and PAI-1 in patients correlate directly with the severity of disseminated intravascular coagulation (DIC) and sepsis. Moreover, these levels are significantly associated with a poor prognosis and a high risk of mortality. In a study involving 107 children with sepsis, Green et al. explored the levels of tissue factor (TF) and PAI-1 antigens. The results indicated that high levels of TF and PAI-1 were related to increased IL-6, cardiovascular and renal abnormalities, liver failure, coagulopathy, and a higher mortality rate (*p* < 0.05) [78].

Another crucial aspect of the hemostasis system is the thrombin-activated inhibitor of fibrinolysis (TAFI) [79,80,81]. The activated form of TAFI can suppress fibrinolysis. This occurs through the removal of the terminal lysine molecule in fibrin, which inhibits the high-affinity binding site for plasmin [82]. The existing literature discusses TAFI gene polymorphisms, but it is still unclear which gene variants are linked to a more severe disease course [83]. The role of TAFI in neonates is poorly understood. Emonts et al. observed a decrease in TAFI levels in patients with septic shock and an increase in TAFI activation peptide (TAFI-AP) in patients with pediatric DIC [84].

The complement system plays a crucial role in the immune response, and its effective neutralization of foreign agents is essential. In sepsis, the complement system fails to adequately respond to infection, leading to the uncontrolled release of chemoattractants C3a and C5a, which bind to receptors on the surface of macrophages and neutrophils [85]. Component C5a activates the coagulation system, specifically factor XII and kallikrein, resulting in thrombosis and the development of DIC. New therapies that target the complement system may effectively combat septic shock and coagulation disorders in children [86].

In addition to the clinical manifestations of DIC, patients with septic shock may show signs of thrombotic thrombocytopenic purpura (TTP). TTP is a condition caused by either a congenital defect or, more commonly, an acquired deficiency of the metalloproteinase ADAMTS-13 (von Willebrand factor-cleaving protease), which primarily functions to cleave vWF multimers into monomeric fragments [87]. This deficiency leads to microvascular obstruction, resulting in organ damage, especially in the brain and kidneys. Additionally, thrombocytopenia and non-immune hemolytic anemia are observed [88]. In addition to overuse, thrombocytopenia (a platelet count of less than 100 × 10^9^/L) in patients with sepsis may arise from either delayed platelet formation or spleen sequestration [89]. The formation of vWF multimers is stimulated by inflammatory mediators such as IL-8 and TNF-α, while the action of ADAMTS-13 is inhibited by IL-6 and antimicrobial peptides secreted by neutrophils [90]. Furthermore, during the inflammatory process, vWF and ADAMTS-13 undergo oxidation, which impairs the cleavage of vWF multimers by metalloproteinases [91].

Several studies indicate that over one-third of patients with sepsis exhibit ADAMTS-13 levels that are two times lower than normal, with approximately 15% of patients showing levels below 10% of normal. A decrease in ADAMTS-13 levels correlates with a heightened risk of mortality in patients facing sepsis and septic shock [92,93,94,95]. Enzyme deficiency leads to the accumulation and prolongation of vWF action, which increases the risk of mortality due to the development of a prothrombotic state in patients [96,97]. Papadogeorgou et al. showed a significant reduction in ADAMTS-13 levels in neonates with sepsis during the acute phase of infection compared to controls (488.5 ± 75.4 ng/mL versus 577.2 ± 113.6 ng/mL, *p* = 0.015) [98].

The role of platelets in the coagulopathy associated with sepsis is significant. During the inflammatory process, P-selectin (an intercellular adhesion protein) is expressed on the platelet surface. P-selectin, located in the α-granules of platelets and the Weibel-Palade secretory granules of endothelial cells, is involved in the primary interaction between polymorphonuclear neutrophils and endotheliocytes. In conjunction with cytokines, it can regulate the synthesis of integrins [99]. Bacteria from the Staphylococcus and Escherichia coli families increase GPIIb/IIIa receptor expression on activated platelet surfaces and activate FcγRIIA [100]. Additionally, platelets help attract other innate immune cells such as granulocytes, monocytes, and innate lymphoid cells (ILCs) [101]. Consequently, platelets play a crucial role in the immunological response to bacterial invasion.

In patients with septic shock, thrombocytopenia is often linked to increased platelet consumption for clot formation [102]. Neonatal thrombocytopenia is more likely to arise from a reduction in the expression of platelet Toll-like receptor 4 (TLR-4), which is associated with a higher mortality rate [103]. A decrease in MyD88 expression on monocytes is also observed in neonates, which correlates with a higher occurrence of severe bacterial infections and septicemia [104]. Furthermore, preterm neonates with histologically confirmed chorioamnionitis demonstrated elevated levels of CD40L expression in platelets compared to the control group (5.3 +/− 2.9% vs. 1.6 +/− 0.7%, *p* < 0.05), underscoring the significant role of platelets in the pathological process [105,106].

### 2.8. Formation of Neutrophil Extracellular Traps and Sepsis

Recently, the role of neutrophils and the formation of neutrophil extracellular traps (NETs), or netosis, in the pathogenesis of sepsis has become increasingly significant. For a considerable time, neutrophils were viewed as a uniform population. However, recent studies have shown that these cells can be subdivided based on their density, surface marker expression, and stage of maturation [107]. NETs consist of modified chromatin and proteins from the cytoplasm, nuclei, and granules of neutrophils, which possess antimicrobial properties. NETs can be triggered not only by microorganisms but also by platelets, immune complexes, complement system proteins, anti-inflammatory cytokines, and other biologically active substances [108]. They create a framework for binding platelets, erythrocytes, and plasma proteins [109]. Platelets can stimulate neutrophils to form NETs. To date, two mechanisms of neutrophil activation have been identified: the first mechanism involves direct contact between an activated platelet and a neutrophil. The platelet TLR-4 plays a crucial role in this process, and some authors have also suggested that pro-inflammatory receptors CXCR4 and CXCR7 are involved in NET formation [110]. The second mechanism occurs through platelets’ release of active molecules in response to factors such as pathogen exposure. Upon platelet stimulation, P-selectin has increased surface expression, which is key in preparing neutrophils for netosis [111]. Conversely, histones present in NETs can activate platelets, creating another link between inflammation and thrombosis [112]. Tissue factor is also an important activator of coagulation. NETs induce TF production via cathepsin G and IL-1α [113]. If NETs contain TF, this system can enhance thrombin generation and PAR-1 signaling in platelets, establishing yet another connection between inflammation and thrombosis. The formation of neutrophil extracellular traps is diminished in preterm neonates, which correlates with a higher incidence of sepsis during the neonatal period [114]. While the formation of NETs reduces the spread of infection, they are also linked to the development of DIC and microvascular damage [115]. Consequently, further research is needed to elucidate the mechanisms in children.

### 2.9. The Damage of the Endothelial Glycocalyx and Increased Vascular Permeability

Additionally, damage to the endothelial glycocalyx can lead to sepsis in children. The glycocalyx plays a crucial role in maintaining vascular homeostasis, regulating vascular permeability and microcirculation, preventing microvascular thrombosis, and controlling leukocyte adhesion [116]. The sympathoadrenal system may become hyperactivated in response to shock, which can subsequently result in damage to endothelial cells and the glycocalyx. The anticoagulation system interacts with the endothelial glycocalyx system through various components, including antithrombin III and activated factors IX and X. The anticoagulant activity of antithrombin III is enhanced through its binding to heparan sulfate, a structural component of the glycocalyx. A potential marker of glycocalyx damage in neonatal sepsis is matrix metalloproteinases (MMP-8, MMP-9). Studies have demonstrated a correlation between MMP-8 and MMP-9 levels, the early onset of sepsis, and an increased risk of multi-organ failure in children [117,118].

### 2.10. The Damage of the Endothelial Glycocalyx and Increased Permeability

Despite the increasing scientific understanding of the pathogenic mechanisms underlying neonatal sepsis, four out of every ten infants born with sepsis either die or experience significant disabilities. Numerous clinical and serum markers have been evaluated for diagnosing sepsis and assessing its severity and cause. From this perspective, the search for early and accurate diagnostic and prognostic biomarkers is beginning to reveal molecules with potential clinical utility, namely microRNAs.

MicroRNAs (miRNAs) are small non-coding RNA molecules that regulate gene expression by binding to messenger RNA (mRNA), which is involved in immune and inflammatory responses as modulators of critical biological pathways and processes [119]. Dysregulated miRNA expression profiles have been identified in sepsis, indicating their potential as both diagnostic and prognostic biomarkers. For instance, specific miRNAs such as miR-155-5p, miR-21, miR-223, miR-146a, and miR-125a have shown promise in detecting sepsis, with pooled sensitivities and specificities ranging from 0.67 to 0.85 and SROC values demonstrating their diagnostic accuracy [120,121].

MiR-146a is known to modulate the immune response by targeting key signaling molecules such as TRAF6 (TNF receptor-associated factor 6) and IRAK1,2 (interleukin-1 receptor-associated kinases 1 and 2), which are involved in the NF-κB pathway. Its upregulation has been observed in septic patients, indicating its role in dampening excessive inflammation responses. Similarly, levels of miR-150 are significantly decreased in sepsis, correlating with disease severity and mortality. This miRNA influences the activation and function of immune cells, thus acting as a potential prognostic marker [119]. MiR-223, another miRNA of interest, regulates granulocyte function and inflammation by targeting various mRNAs involved in these processes. Its expression is dysregulated in septic shock, making it a viable candidate for both diagnostic and prognostic applications.

Unlike traditional diagnostic methods such as leukocytosis and fever monitoring, miRNAs offer a more specific and stable biomarker profile. Their presence in circulating blood makes them accessible for non-invasive testing, potentially leading to earlier and more accurate diagnosis of septic shock [122]. This novel approach not only aids in early detection but also provides insights into the molecular mechanisms underlying sepsis, paving the way for targeted therapeutic interventions.

### 2.11. The Role of the Convergent Model of Coagulation in Septic Shock

**Figure 3:**
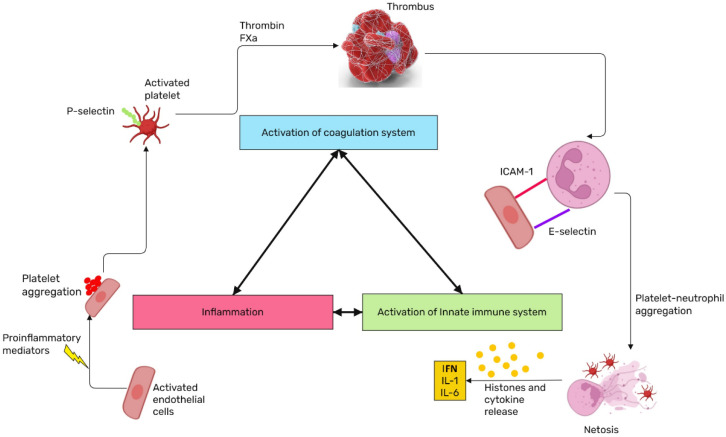
The concept of the coagulation convergent model. Coagulation is closely integrated with inflammation and innate immunity, forming a unified response to vascular injury beyond traditional cascade and cell-based concepts. IL (Interleukin); FXa (Blood coagulation factor Xa); ICAM-1 (Soluble intercellular adhesion molecule-1); IFN (Interferon).

The convergent model of coagulation represents a paradigm shift in our understanding of hemostasis and thrombosis, integrating inflammation and innate immune activation as a unified response to vascular injury [123] (Figure 3). This model builds on previous advancements in our knowledge of the coagulation cascade by incorporating the role of damage-associated molecular patterns (DAMPs), which facilitate interactions within and between systems to reinforce and resolve clot formation. The aim of this model is to extend the boundaries of coagulation to address innovative diagnostics and therapeutics for contemporary medical challenges, notably highlighted by the COVID-19 pandemic and vaccine-induced immune thrombotic thrombocytopenia. Upon vascular injury, the coagulation cascade is initiated, activating various immune cells such as platelets, monocytes/macrophages, and neutrophils. Platelets not only play a role in clot formation but also release cytokines and chemokines that attract additional immune cells to the site of injury, thereby strengthening the immune response. Monocytes and macrophages contribute by expressing tissue factor (TF), a critical initiator of the extrinsic coagulation pathway, thereby further linking coagulation with immune response activation. Neutrophils, another key component, release extracellular traps (NETs) that provide a scaffold for clot formation and capture pathogens, simultaneously enhancing both coagulation and immune defense. This integrated response highlights the complex yet coordinated interactions between the coagulation system and innate immunity in maintaining hemostasis and defending against infections [124].

## 3. The Early Diagnosis and Treatment of Septic Shock

In line with the recommendations of international guidelines, a systematic screening process for sepsis and septic shock is warranted when acute multiorgan failure is identified [125]. The prompt identification of sepsis and septic shock facilitates the administration of appropriate treatment. The diagnosis of sepsis primarily relies on isolating the pathogen in blood cultures. Therefore, it is crucial to isolate the pathogen before initiating antibiotic therapy. Additionally, distinguishing between septic shock and other types of shock, which may present similar symptoms, is essential. Lactate serves as a nonspecific marker of septic shock, indicating the presence of tissue hypoperfusion. However, measuring blood lactate is not recommended according to international guidelines.

**Figure 4:**
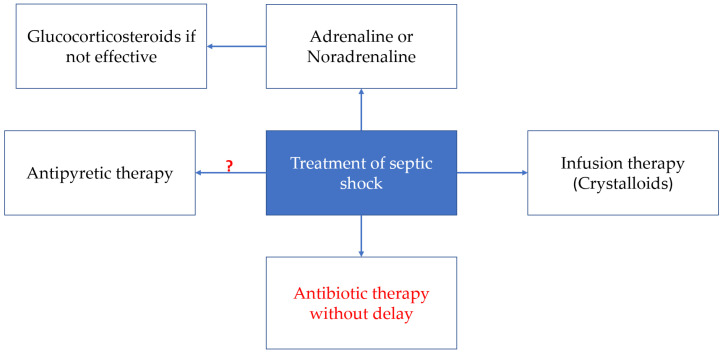
Septic shock treatment strategies.

The treatment of septic shock encompasses a range of interventions, including cardiovascular and respiratory support (Figure 4). In the event of cardiovascular damage, infusion therapy or inotropic support is required [126]. If the neonate exhibits symptoms indicative of septic shock, including cyanosis, marbling, and a weakened pulse wave, it is recommended to start treatment with prostaglandins [127]. In 2020, Weiss et al. published the international guidelines for the management of septic shock and organ dysfunction associated with sepsis in children. These guidelines include the following treatment steps [125].

### 3.1. Antimicrobial Therapy

It is recommended that antibiotic therapy be initiated promptly in neonates showing initial signs of septic shock. However, in children experiencing multi-organ failure due to sepsis but not exhibiting symptoms of septic shock, antimicrobial therapy is also recommended. Selecting a broad-spectrum antibiotic with a wide therapeutic window is preferable to minimize the risk of undesirable side effects. Discontinuation of antibiotic therapy is possible if it proves ineffective, if side effects occur, or if no isolated pathogen is identified. Intravascular access is not recommended, as catheters can act as an additional source of infection and may also complicate the course of septic shock, prolonging the recovery period [128]. The high mortality rate of neonates with septic shock results from inappropriate or delayed antibiotic therapy. The safest medications should be prescribed, considering all pathological symptoms of the neonate and the risk–benefit ratio for each patient based on individual circumstances. Third-generation cephalosporins, such as ceftriaxone, may serve as an initial therapeutic option for out-of-hospital sepsis. If there is antibiotic resistance to cephalosporins, it is essential to adjust the approach to antibiotic therapy and prescribe a drug from the aminoglycoside class [129]. The greatest challenge in managing sepsis and other serious infections caused by antibiotic-resistant bacteria, such as Methicillin-resistant Staphylococcus aureus (MRSA), is the lack of effective therapeutic options. In these cases, vancomycin is often added to the treatment regimen to enhance its efficacy.

### 3.2. Infusion Therapy

Infusion therapy to correct the hypovolemia caused by septic shock is recommended as 40–60 mL/kg bolus (10–20 mL/kg bolus) during the first hour. Concurrently, both BP and pulse control should be carried out. If signs of fluid overload develop, infusion therapy should be discontinued. It is recommended that infusion therapy be replaced with crystalloids, as they have fewer side effects, rather than albumin and physiological solution. Starch (e.g., Hydroxyethyl Starch) is not recommended as a treatment for septic shock in children. In a study involving adult patients with septic shock, starch increased the risk of death, coagulopathy, and acute kidney injury [130]. Measuring lactate levels is not recommended as a marker of septic shock in children. However, they can be used to assess the adequacy and effectiveness of infusion therapy [131]. Elevated lactate levels may indicate incomplete or inadequate hemodynamic resuscitation, which necessitates continuing infusion therapy or increasing the bolus fluid volume.

### 3.3. Vasoactive Drugs

International guidelines recommend using adrenaline or noradrenaline instead of dopamine as the first-line therapy for children with septic shock. If signs of impaired perfusion are observed after infusion therapy, administering vasoactive drugs via intravenous or intraosseous access (when available) should be initiated. A comparison of the efficacy of adrenaline and dopamine has shown that adrenaline therapy reduces mortality, unlike dopamine. (OR, 0.63; 95% CI, 0.40–0.99) [132,133].

### 3.4. Corticosteroids

According to international clinical guidelines, glucocorticosteroids are acceptable if infusion therapy and treatment with vasoactive drugs prove ineffective. A study demonstrated the efficacy of glucocorticosteroid therapy in children, showing that the administration of these medications led to earlier relief from septic shock (*p* = 0.046) [134]. Furthermore, there are no more extensive or detailed studies in the pediatric population regarding the use of hydrocortisone in the treatment of septic shock.

### 3.5. Antipyretic Therapy

There is no consensus among healthcare professionals regarding the use of antipyretics in children. However, international guidelines do recommend their use in patients with septic shock. Currently, there is no clear and uniform protocol for managing neonatal septic shock in infants. Treatment depends on several factors, including the child’s age and weight, the presence or absence of nonspecific symptoms, comorbidities, and the genetically determined response to drug therapy. Primarily, therapy for septic shock should focus on saving the patient’s life, alleviating adverse symptoms of the underlying illness, and minimizing undesirable side effects of drug therapy. International guidelines offer a general framework for the approach to therapy for septic shock.

## 4. Conclusions

The impact of FIRS and septic shock on the newborn organism is significant, affecting all systems. The fetal systemic inflammatory response is considered an adaptive mechanism. However, it can become dysregulated, leading to a fetal cytokine storm, resulting in multiple organ dysfunction and even fetal death. It is important to consider the changes occurring in the still-immature hemostatic system. The nonspecific clinical features of septic shock, characterized by damage to various body systems (from cardiovascular to endocrine), present a significant challenge for clinicians. The clinical manifestations in newborns differ significantly from those in adults due to the organism’s unique characteristics. Administering antimicrobial agents, anti-inflammatory and vasoactive drugs, glucocorticosteroids, and infusion therapy may help modulate a dysregulated fetal inflammatory response, potentially decreasing infant morbidity and mortality. A deeper understanding of the underlying pathogenic mechanisms will facilitate the development of more effective therapeutic approaches and improve the prognosis for these children.

## Figures and Tables

**Table 1:** Diagnostic criteria for SIRS.

Criterion	Value
Body temperature (rectal, oral)	>38.5 °C or <36 °C
Heart rate	Tachycardia ≥ 90/min, or bradycardia in children under 1 year of age below the 10th percentile
Respiratory rate	≥20/min or hyperventilation with blood carbon dioxide ≤ 32 mmHg
White blood cell count	Leukocytosis or leukopenia or neutrophil left shift

**Table 2:** Potential risk factors for the development of sepsis.

Maternal risk factors	Age over 30 years Chorioamniotis Premature labor Presence of meconium in amniotic fluid Rectovaginal colonization with group B streptococcus. Infectious diseases (urinary tract infection) and intrapartum fever
Fetal risk factors	Central venous catheter placement Prematurity (less than 37 weeks of age) Low birth weight (less than 2500 g) Apgar score less than 5 points Artificial ventilation Lack of enteral nutrition Pathology of the gastrointestinal tract Hemodynamic disorders (neutropenia and decreased serum IgG concentration)
